# Contaminated Sites in Europe: Review of the Current Situation Based on Data Collected through a European Network

**DOI:** 10.1155/2013/158764

**Published:** 2013-06-16

**Authors:** Panos Panagos, Marc Van Liedekerke, Yusuf Yigini, Luca Montanarella

**Affiliations:** European Commission, Joint Research Centre, Institute for Environment and Sustainability, Via E. Fermi 2749, 21027 Ispra, Italy

## Abstract

Under the European Union (EU) Thematic Strategy for Soil Protection, the European Commission has identified soil contamination as a priority for the collection of policy-relevant soil data at European scale. In order to support EU soil management policies, soil-related indicators need to be developed which requires appropriate data collection and establishment of harmonized datasets for the EU Member States. In 2011-12, the European Soil Data Centre of the European Commission conducted a project to collect data on contaminated sites from national institutions in Europe using the European Environment Information and Observation Network for soil (EIONET-SOIL). This paper presents the results obtained from analysing the soil contaminated sites data submitted by participating countries. According to the received data, the number of estimated potential contaminated sites is more than 2.5 million and the identified contaminated sites around 342 thousand. Municipal and industrial wastes contribute most to soil contamination (38%), followed by the industrial/commercial sector (34%). Mineral oil and heavy metals are the main contaminants contributing around 60% to soil contamination. In terms of budget, the management of contaminated sites is estimated to cost around 6 billion Euros (€) annually.

## 1. Introduction

Soil contamination creates a significant risk to human health. For instance, heavy metals from industrial waste contaminate drinking water, soil, fodder, and food [1]. Also, the large volume of waste and the intense use of chemicals during past decades have resulted in numerous contaminated sites across Europe. Contaminated sites could pose significant environmental hazards for terrestrial and aquatic ecosystems as they are important sources of pollution which may result in ecotoxicological effects [2].

Emissions of hazardous substances from local sources could deteriorate soil and groundwater quality. Management of contaminated sites aims at assessing the adverse effects caused and taking measures to satisfy environmental standards according to current legal requirements. Additionally, the impact of soil contamination to health and more specifically the main epidemiological findings relevant to CS are briefly presented below.

The implication of soils to human health is direct such as ingestion, inhalation, skin contact, and dermal absorption. Some epidemiological examples include geohelminth infection and potentially harmful elements via soil ingestion, cancers caused by the inhalation of fibrous minerals, hookworm disease, and podoconiosis caused by skin contact with soils [3]. Elliott et al. (2001) [4] have found small excess risks of congenital anomalies and low and very low birth weights in populations living near landfill sites.

Soil contamination is mainly located close to waste landfills, industrial/commercial activities diffusing heavy metals, oil industry, military camps, and nuclear power plants. As European society has grown wealthier, it has created more and more rubbish. Each year in the EU, 3 billion tonnes of solid wastes are thrown away (some 90 million tonnes of them are hazardous). This amounts to about 6 tonnes of solid waste for every man, woman, and child (Eurostat, Environmental Data Centre on Waste [5]).

The main anthropogenic sources of heavy metals exist in various industrial point sources, for example, present and former mining activities, foundries, smelters, and diffuse sources such as piping, constituents of products, combustion of by products, and traffic related to industrial and human activities [6].

In the US, the army alone has estimated that over 1.2 million tons of soils have been contaminated with explosives, and the impact of explosives contamination in other countries in the world is of similar magnitude [7]. In recent years, growing concerns about the health and ecological threats posed by manmade chemicals have led to studies of the toxicology of explosives, which have identified toxic and mutagenic effects of the common military explosives and their transformation products [8]. Papp et al. (2002) [9] have studied the significant radioactive contamination of soil around a coal-fired thermal power plant.

Different contaminants have different effects on human health and the environment depending on their properties. The contaminant effect depends on its potential for dispersion, solubility in water or fat, bioavailability, carcinogenicity, and so forth. Chlorinated hydrocarbons (CHCs) are used mainly for the manufacturing of synthetic solvents and insecticides. They are environmental contaminants that bioaccumulate and hence are detected in human tissues. Epidemiological evidence suggests that the increased incidence of a variety of human cancers, such as lymphoma, leukemia, and liver and breast cancers, might be attributed to exposure to these agents [10].

Mineral oil large-scale use and various applications lead in many cases to environmental contamination [11]. Such contamination may be a consequence of petroleum transport, storage and refining, or accidents [12]. From a quantitative perspective, mineral oil is probably the largest contaminant in our body. That humans can tolerate this contaminant without health concerns has not been proven convincingly. The current Editorial of the European Journal of Lipid Science and Technology concludes that this proof either has to be provided or we have to take measures to reduce our exposure (from all sources, including cosmetics and pharmaceuticals) and the environmental contamination.

Polycyclic aromatic hydrocarbons (PAHs) are semivolatile, chemically stable, and hydrophobic organic compounds which are ubiquitous in the environment and good markers of urban activities. PAHs are related with anthropogenic toxic element contamination [13].

Heavy metals have been used by humans for thousands of years. Although several adverse health effects of heavy metals have been known for a long time, exposure to heavy metals continues and is even increasing in some parts of the world, in particular in less developed countries, though emissions have declined in most developed countries over the last 100 years [14]. Any metal (or metalloid) species may be considered a “contaminant” if it occurs where it is unwanted, or in a form or concentration that causes a detrimental human or environmental effect. Metals/metalloids include lead (Pb), cadmium (Cd), mercury (Hg), arsenic (As), chromium (Cr), copper (Cu), selenium (Se), nickel (Ni), silver (Ag), and zinc (Zn). Other less common metallic contaminants include aluminium (Al), cesium (Cs), cobalt (Co), manganese (Mn), molybdenum (Mo), strontium (Sr), and uranium (U) [15].

According to WHO, priority should be given to the pollutants on the basis of toxicity, environmental persistence, mobility, and bioaccumulation [16]. Many of the heavy metals such as cadmium, arsenic, chromium, nickel, dioxins, and PAHs are considered to be carcinogenic, based on animal studies or studies of people exposed to high levels [17]. In addition to carcinogenicity, many of these substances can produce other toxic effects (depending on exposure level and duration) on the central nervous system, liver, kidneys, heart, lungs, skin, reproduction, and so forth.

The toxicity and fate of phenolic pollutants in the contaminated soils are associated with the oil-shale industry [18]. Phenol has been shown to cause liver and kidney damage, neurotoxic effects, and developmental toxicity in laboratory animals (Environment Agency, 2009).

The most common source of cyanide contamination is former gas work sites. However, cyanide contamination is also associated with electroplating factories, road salt storage facilities, and gold mining tailings [19]. Cyanide toxicity results from inhibition of cytochrome oxidase thereby limiting the absorption of oxygen at the cellular level. The central nervous system is a major target of acute cyanide toxicity, with a short period of stimulation evidenced by rapid breathing, followed by depression, convulsions, paralysis, and possibly death [20].

Benzene, toluene, ethylbenzene, and xylene (BTEX) are classified as hazardous air pollutants (HAPs) [21]. Exposure to HAPs can cause a variety of health problems such as cancerous illnesses, respiratory irritation, and central nervous system damage [22].

The objective of relevant EU policies is to achieve a quality of the environment where the levels of manmade contaminants on sites do not give rise to significant impacts or risks to human health and ecosystems. The most recent developments in soil policy at European level are the introduction of the thematic strategy for the protection of soils [23] and the proposed soil framework directive [24]. Soil contamination is recognised as one of the eight soil threats expressed in the thematic strategy and the proposed directive. As there was not a consensus for the establishment of the soil framework directive, legal requirements for the general protection of soil have not been agreed at EU level and only exist individually in most Member States. However, the integrated pollution and prevention control directive [25] requires that operations falling under its scope do not create new soil contamination. Other EU directives such as the water framework directive [26] and the waste directive [27], not aimed directly at soil protection, provide indirect controls on soil contamination [28]. Notwithstanding these controls, some significant new site contamination still occurs as a result of accidents [29] and illegal actions. While the creation of new contaminated sites is constrained by regulation, a very large number of sites exist with historical contamination that may present unacceptable risks and these sites require management. One example is the environmental disaster following flooding by red sludge in the Ajka region in Hungary [30]. However, the research and political arena regarding land contamination no longer consider only a few incidents that lead to severe soil contamination, but rather look at it as a wide spread environmental problem.

In 2001, the European Environment Agency (EEA) in cooperation with EEA affiliated countries started to develop a core set of policy relevant indicators, among which the indicator “Progress in the Management of Contaminated Sites” (CSI015) was the only one related to soil. Since then, data collections in relation to this indicator were launched four times by EEA [31], the last one in 2006, with contribution from member countries of the European Environment Information and Observation Network (EIONET) [32]. In the period 2011-2012, the European Soil Data Centre (ESDAC) [33] organized a similar campaign in order to update the CSI015. This indicator quantifies the progress in the management of local contamination, identifies sectors with major contribution to soil contamination, classifies the major contaminants, and finally addresses issues of budgets spent for remediation. The indicator is very important for policy makers as it tracks progress in the management of contaminated sites and the provision of public and private money for remediation. With this indicator, a number of activities causing soil pollution can be clearly identified across Europe. The indicator also supports the implementation of existing legislative and regulatory frameworks (integrated pollution prevention and control directive, landfill directive, water framework directive) as they should result in less new contamination of soil.

The present study presents an overall picture in Europe concerning contaminated sites and does not focus on individual countries. Instead, there are many other studies, such as the one of Ferguson (1999) [34], that present the inventories of contaminated sites for individual countries. The overall objective of this paper is to make an overview of the current situation of contaminated sites in Europe. Specifically, the study intends to

- focus on contaminated sites caused by industrial activities;
- review the type of sources;
- respond to the main policy questions addressed in the indicator CSI015.

## 2. Materials and Methods

The study makes an assessment of the data collected through EIONET and then focuses on the data related to contamination as a consequence of industrial activities.

### 2.1. EIONET-CSI Data

The contaminated sites data (denoted as EIONET-CSI from now on) were collected and managed by the European Soil Data Centre (ESDAC). The data were collected in 2011-2012 through the EIONET network which consists of representative organizations from 38 European countries for a number of environmental themes [35]. The appointed organisations for the theme “soil” are lead institutions in the soil domain at national level, and they provide official country data on specific requests related to soil by ESDAC.

The geographical coverage of EIONET includes 27 Member States of the European Union together with Iceland, Liechtenstein, Norway, Switzerland, Turkey, and the West Balkan cooperating countries: Albania, Bosnia, Herzegovina, Croatia, the former Yugoslav Republic of Macedonia, Montenegro, and Serbia as well as Kosovo under the UN Security Council Resolution 1244/99. Similar data on contaminated sites have been collected in 2001, 2002, 2003, and 2006. The data were collected through a standard questionnaire and then compiled in a centralized database. The questionnaire was designed such that received data could feed the compilation of the indicator, the CSI015 indicator. There is no legal obligation for the EIONET member countries to submit data, and their contribution is on a voluntary basis.

### 2.2. Terms and Definitions

In order to minimize the differences in interpretation by individual countries of certain terms used in the questionnaire, ESDAC provided the following definitions according to EEA [31].

- “Contaminated site” (CS) refers to a well-defined area where the presence of soil contamination has been confirmed and this presents a potential risk to humans, water, ecosystems, or other receptors. Risk management measures (e.g., remediation) may be needed depending on the severity of the risk of adverse impacts to receptors under the current or planned use of the site.
- “Potentially contaminated site” (PCS) refers to sites where unacceptable soil contamination is suspected but not verified, and detailed investigations need to be carried out to verify whether there is unacceptable risk of adverse impacts on receptors.
- “Management of contaminated sites” aims to access and, where necessary, reduce to an acceptable level the risk of adverse impacts on receptors (remediate). The progress in management of CS is traced in 4 management steps starting with preliminary study, continuing with preliminary investigation, followed by site investigation, and concluding with implementation of site remediation (reduction of risk).

There is an important definition in terminology which allows the readers of the article to distinguish between “estimated” and “identified” sites. The questionnaire asked the countries to provide estimations on how many CSs and PCSs may be situated in their territory. Data on estimated CS and PCS is based on studies or expert judgment. The questionnaire also asked for identified number of CS and PCS. In this case, the countries report data for which they actually posses available information about local soil properties and hydrology.

### 2.3. Other Datasets

For a more comprehensive assessment, a number of auxiliary official Eurostat datasets [35] were used such as the countries' populations, the surface area, the gross domestic product (GDP), and the number of enterprises in the industrial/services sectors. Those datasets are used for developing statistics with parameters that include the surveyed population, the surveyed area, the density of CS and PCS, the contribution (%) of various industrial sectors to contamination, and the proportion of budget spent for management of CS.

### 2.4. Methodology

The study is based on the received data from the countries that participated in the survey, replying to the questionnaire available in the European Soil Portal [36]. The questionnaire has a user-friendly format as a Microsoft Excel file and contains 5 main sections: “management of contaminated sites,” “contribution of polluting activities to local soil contamination,” “environmental impacts,” “expenditures,” “remediation targets and technologies.” Each section includes between 1 and 5 questions requesting the “user” to submit the data for each of the available options. The questionnaire was requesting numerical values (not classes or vague responses) which allowed making aggregations depending on the policy question that was to be addressed. Two example questions are the following: percentage (%) of sites, where risk reduction measures are completed; expenditures in million euro per capita per year. As a support, a guidelines document was available with detailed explanation for each of the questions and the possible options plus example responses based on previous data collection exercises.

Each country represented by its designated EIONET National Reference Centre for soil provided its best assessment based on available data. The data collection campaign was launched in October 2011 and ended in February 2012.

## 3. Results 

Even if the questionnaire included other data and information, this paper mainly focuses on the local contamination analysis, the type of contamination (which sectors are contributing the most), the distribution of the main contaminants, and the budget spent for remediation. The management of CS will not be analysed in detail as each country follows a different approach concerning the management steps. The analysis is performed in the study area as a whole and not at country level. It should be noted that quite different interpretations of the abovementioned definitions have been applied by individual countries.

### 3.1. Extent of Local Contamination in Europe

**Figure 1:**
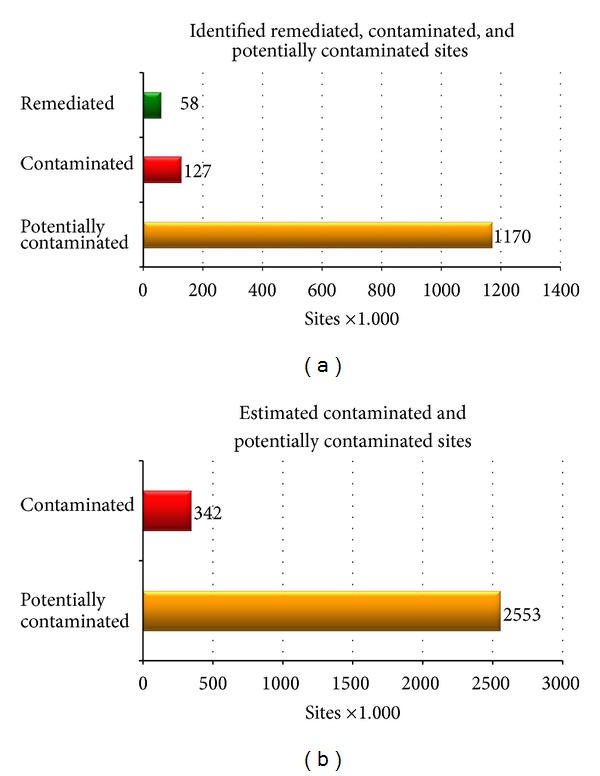
(a) Number of identified remediated (RS), potentially (PCS), and contaminated Sites (CS) reported by 33 countries. (b) Number of estimated potentially (PCS) and contaminated sites (CS) extrapolated to 38 countries.

Data on soil contamination per country is a necessary input in order to estimate the scale of soil contamination in Europe. The majority of the addressed countries (33 out of 38 countries), corresponding to 80% of the total population, have responded with data on the identified number of PCS and CS (Figure 1(a)). The missing five countries were Bosnia, Herzegovina, Poland, Portugal, Slovenia, and Turkey. According to Figure 1(a), around 1,170,000 PCSs have been identified in Europe till 2011. More than 10% of them or around 127,000 have been identified or confirmed as CSs. The ratio of remediated sites (RSs) to CSs is around 45% as more than 58,000 CSs have already been remediated (Figure 1(a)). The data gap for the 5 missing countries can be covered by employing the density of PCS (2.4 PCS/1,000 capita) and CS (2.62 CS/10,000 capita) (Table 1). Applying the average of 2.4 identified PCS per 1,000 capita for the 5 missing countries, the identified PCS for the whole Europe (38 countries) is estimated to be around 1,470,000. Applying the average of 2.62 identified CS per 10,000 capita (Table 1, column (a)) for the missing 5 countries, the identified CS can be raised to 160,000.

Apart from the identified PCS and CS, countries have been asked to provide their estimations for those 2 figures. A subset of 12 countries out of the 33 participating ones has provided estimations about the PCS (Table 1, column (b)). As a rule of thumb, the estimations are greater than the identified ones. According to their estimations, 740,000 PCSs may exist in their territory with a density of 4.2 PCS/1,000 capita. Those 12 countries have reported 520,000 PCSs which result that the ratio “*identification to estimation*” for PCS is around 70%. Two types of extrapolations can be performed in order to estimate the total number of PCS. In the first one, the average value of 4.2 PCS/1,000 capita is applied to the total population of the 38 countries, and the total number of estimated PCS is then around 2,553,000 (Figure 1(b)). In the second extrapolation method, the ratio “*identification to estimation”* for PCS (70%) is applied to the countries which were unable to provide estimations; then the approximate number of PCS can be estimated to be 2,087,000.

Another subset of 11 countries (not a sub group of the previous 12) covering 10% of total population has provided estimations about CS. They estimated more than 32,000 CSs with a density of 5.7 CS/10,000 capita (Table 1, column (c)). Those 11 countries have reported 10,036 identified CSs which result that the ratio “*identification to estimation*” for CS is 30.7%. The first method of extrapolation is to apply the average density to the rest of the population (90%), where data does not exist. According to this estimation, the number of CS in Europe is estimated to be around 342,000 which accounts for 14% of the total estimated PCS (Figure 1(b)). In the second extrapolation method, the ratio “*identification to estimation*” for CS (30.7%) is applied to the whole population, and the estimated number of CS becomes more than 516,000. When comparing to the last survey of 2006, the estimated number of PCS was around 3 million, and the estimated number of CS was around 250,000.

**Figure 2:**
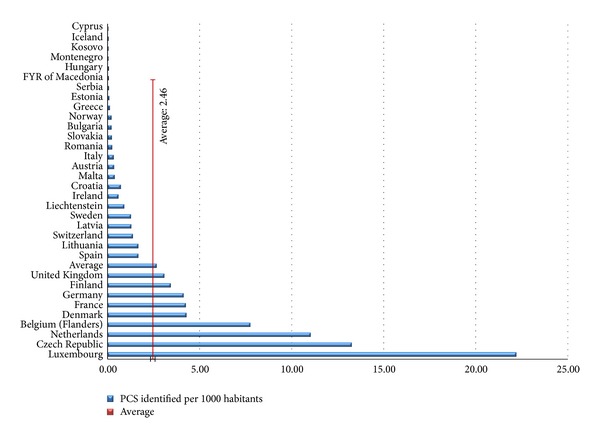
Density (identified PCS/1,000 capita) in 33 countries.

The high variability of the data reported can be seen in Figure 2. The huge differences in the density rates represent the situation of PCS per country and how countries interpret the term of “potential contamination.” Interpreting the metadata that come with the received data, PCSs are understood in a different way. For instance, Luxemburg, Belgium, Netherlands, and France include potentially polluting activities in their PCS figures, and this is the reason for high density of PCS in those countries (Figure 2). Other countries such as Austria, Hungary, and Norway include in their PCS figures only the sites where there is an evidence of potential contamination. Another factor contributing to this high variability is the granularity of a site. Some countries report sites which are important at national level, while others include also small sites such as storage tanks.

### 3.2. Sectors Contributing Most to Soil Contamination

Soil contamination is the result of various sectors and activities. The countries were asked to allocate a percentage of contribution of each sector to local soil contamination based on the occurrence of incidents. The following seven categories of activities were proposed:

- waste disposal (municipal waste disposal and industrial waste disposal).
- industrial and commercial activities (mining, oil extraction and production, and power plants).
- military (military sites and war affected zones).
- storages (oil storage, obsolete chemicals storage, and other storages).
- transport spills on land (oil spill sites and other hazardous substance spills sites).
- nuclear.
- other sources.

Responses related to contributing sectors were received from 22 countries which correspond to circa 53% of the total study population. Waste disposal and treatment contribute to more than 37% of soil contamination. Inside this category, municipal waste and industrial waste contribute to similar shares. The industrial and commercial activities contribute to 33.3% share, followed by storage (10.5%), while of the rest have a contribution of 19.1%. Nuclear operations contribute only 0.1%, but contamination from major nuclear players (e.g., scores from nuclear power stations) was not taken into account by some countries. The data cannot be compared to 2006 survey as the sample of countries that responded is dissimilar.

**Figure 3:**
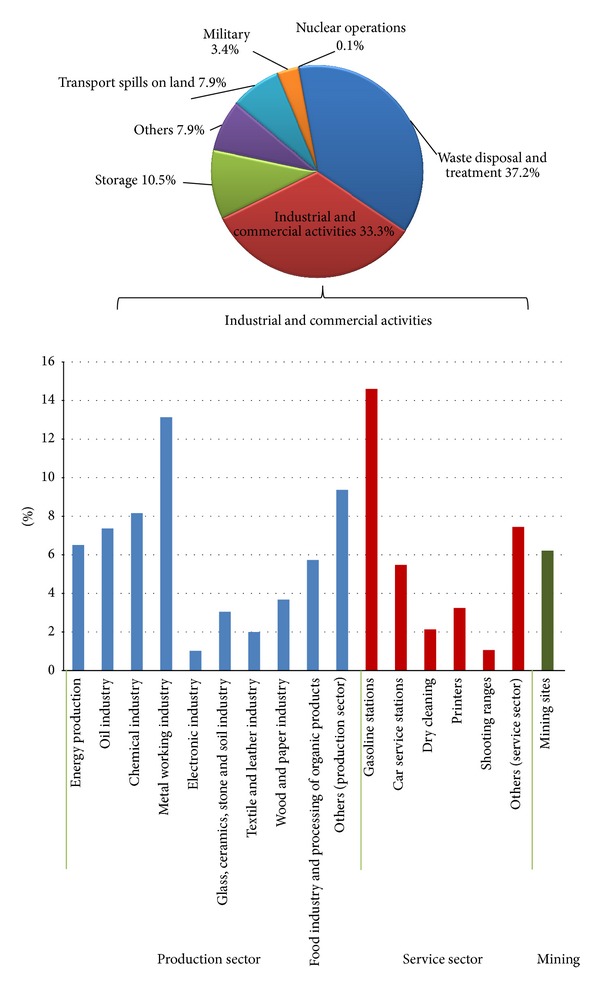
Distribution of sectors contributing to soil contamination in Europe with special focus to industrial/commercial activities.

A special focus is given to the industrial and commercial sectors causing soil contamination. The countries were asked to assign percentages in each specific industrial sector which contributes to soil contamination. The responses of 17 countries covering 44% of the total study population suggested that the production sector contributes to around 60% of soil contamination, while the service sector has a share of 33% and the mining sector contributes to around 7% (Figure 3).

A closer look at the production sector reveals that the textiles, leather, wood, and paper industries are of minor importance for local soil contamination (circa 5%), while metal industries are most frequently reported to be important sources of contamination (13%) followed by chemical industry (8%), oil industry (7%), and energy production (7%) that sum up the 35% of the production sector, while all of the rest (25%) are distributed in 6 categories. For the service sector, gasoline stations are the most frequently reported sources of contamination (15%) followed by the car service stations (around 6%).

The Eurostat data on sectoral breakdown of manufacturing (NACE [37]) sums up the total number of enterprises in the EU to 2.041 million. The Eurostat industrial sectors do not correspond one-to-one with the industrial production sectors considered in the EIONET-CSI questionnaire (Table 2, column (a)). Some grouping of the Eurostat sectors (plus sign in column (c)) has taken place to make the correspondence. Note that the Eurostat data for the mining sector was embedded in the Eurostat category “other manufacturing.” From the values in columns d and b, a new value (column (e)) is computed that expresses how many enterprises of an industrial sector contribute to 1 percent of the local contamination coming from that sector. The smaller the number, the more one site contributes to industrial contamination. The resulting figures show for instance that mining sites are individually heavier polluters compared to other sectors. Instead, the electronic industry enterprises pollute less compared to the shown sectors (Table 2).

### 3.3. Main Contaminants

The countries were asked to allocate a percentage for the proposed contaminant categories based on the occurrence of soil contamination. Distinctions were made between contaminants affecting the solid matrix (soil, sludge, and sediments) and the liquid matrix (groundwater, surface waters, and leachate). The following eight categories of contaminants were proposed both for solid and liquid matrices:

- chlorinated hydrocarbons (CHCs).
- mineral oil.
- polycyclic aromatic hydrocarbons (PAHs).
- heavy metals.
- phenols.
- cyanides.
- aromatic hydrocarbons (BTEX: benzene, toluene, ethyl benzene, and xylene).
- others.

The responses were received from 16 countries which correspond to about 40% of the total study population. The analysis based on these responses is of key importance for research and development, the remediation market, and related industries. For instance, if a specific compound is known to be a major soil contaminant, it may be worthwhile to develop new detection methods (i.e., in situ detection) and more efficient remediation techniques.

**Figure 4:**
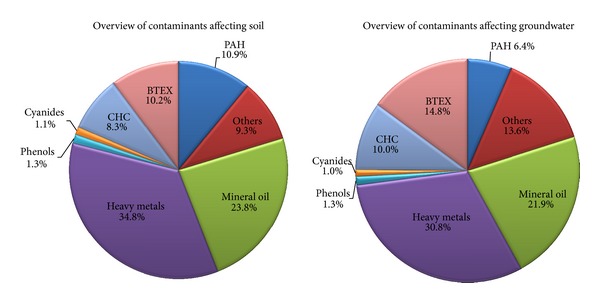
Distribution of contaminants affecting soil and groundwater in Europe.

The distribution of the contaminants affecting soil is similar to the one of groundwater. The main contaminant categories are heavy metals and mineral oil contributing jointly to around 60% in soil contamination and 53% of the groundwater contamination (Figure 4). On the contrary, the phenols and cyanides have an insignificant contribution to total contamination. The remaining four categories (BTEX, CHC, PAH, and others) have similar contributions to soil contamination varying between 8 and 11% and summing up to 40%. In the groundwater contamination, their contribution is around 45% ranging from 6% for PAH to 15% for BTEX. The current distribution is similar to the one proposed after the analysis of the 2006 surveyed results.

### 3.4. Budget Allocated

The cost of managing the CS is an important element taken into account by policy makers. The questionnaire included parts to investigate annual estimation of expenditures, share of private/public money, and share of total expenditure. This is a very important aspect as one of the most criticised issues in the proposed European soil framework directive [24] was the required estimate of annual cost for management of CS. According to the impact assessment of the proposed directive, there was a wide-ranging estimate from 2.4 to 17.3 billion Euros.

According to the responses of 11 countries covering 23% of the total population (139 million out of the 612 million inhabitants for the total area), 1,483.2 million euros (€) were spent annually for the management of CS in these countries. In absolute terms, this is around 10.7€ per capita or 0.041% of the gross domestic product (GDP) for the 11 countries. The reported data show a small decrease in expenditure for management of CS compared to 2006 (12€ per capita).

If this sample of 11 countries is considered representative for the whole Europe, then the management of contaminated sites can be estimated to be 6,526 million euros (€) per year. Compared to the impact assessment of the proposed soil framework directive, this amount of money is probably a more precise estimate of the cost of the management of all identified CSs (including remediation).

Regarding the share of private/public money, 42% of the total expenditure comes from public budgets while the 58% from private investments. Another interesting aspect of the study is the share in the total expenditures for the management of CS for the different management steps. The vast majority (80.6%) is spent for the remediation measures while 15.1% is spent in site investigation and only 4.3% in after care measures and redevelopment of the sites. When considering the budget spent on remediation and the number or remediated sites (RSs) in the 11 reported countries, it is calculated that the average amount spent per RS annually is around 37.1 thousand euros (€) in a range varying from 7.5 thousand € to 232 thousand € annually. As the remediation of sites has a duration of more than 1 year, the majority (40%) of the reported remediation projects fall in the range 50,000 to 500,000€, while a considerable 26.5% of the reported cases fall in the range between 5,000 and 50,000€.

## 4. Discussion and Conclusions 

In terms of estimations, around 1,170,000 PCSs have been identified which are circa 45% of the total estimated PCSs. Also, around 127,000 CSs have been already identified which are circa 27% of the total estimated 342,000 CSs. Moreover, around 46% of the total identified CSs have been remediated (58,300 RSs). The identified figures for CS, PCSs and RS are based on reported data from 33 countries, while the estimated CSs and PCSs have been extrapolated based on data from a limited sample (11 or 12 countries).

Notwithstanding the positive outcomes of the EIONET-CSI data collection, it could be noted that the data submitted were not homogeneous since there are differences in the way that countries interpret the terms of contaminated sites. As shown in Figure 2, there is a high variability between the data submitted by countries. This variability is explained by the large uncertainty both in terms of methodology and data. Some countries run their own CS management system which may not fit perfectly to the definition of the CSI015 indicator, and this contributes to methodology uncertainty. Moreover, the reported data are usually based on expert judgement which includes a high degree of uncertainly. The countries may interpret the data specifications in different ways, and this increases the heterogeneity in the data reported. The reported data on CSI015 indicator are based on the exceed of limits in concentrations of hazardous chemicals. However, common limits are unlikely to be established at the European level since they may be strongly influenced by local soil and geological properties.

An adequate response to the high data variability could be to make a pan-European training event with the participation of competent national EIONET authorities, with the objective to apply the same terminology in all countries in subsequent data collections. The heterogeneity of responses can also be decreased if the provided documentation is taken into account.

In general, there are difficulties in getting the data on soil contamination, but improvement in data availability and data quality over the years can be observed. At this moment, the resulting dataset is the best “picture” that can be achieved based on national data. The EIONET-CSI data collection has taken place 5 years after the previous one of 2006. This 5-year period between data collections seems to be more appropriate than the 2-year period applied in the past, since the data on CS are not changing considerably in such a relative short time.

The direct and indirect costs to a country for dealing with the problem of CS depend on the amount and characteristics of CS in its territory. Generally, the presence of CS can affect company profits, business confidence, and attractiveness to investors. It may also affect aspects of public health and ecosystem protection. The remediation cost of CS, even if only a very little percentage of GDP, seems to be a major issue, and investments to improve the land quality through remediation are not readily made. Countries should weigh the costs of dealing with local land contamination against benefits to public health, improvement of the environment (e.g., water quality), land regeneration, and sustainable use of soil.

Restrictions set by privacy law in Europe are a major obstacle to identification and management of land contamination. Status and data on private land are not easily accessible to public authorities as this may have some implications for the land owner. However, the situation of his land is affecting public health, water quality, and ecosystem services. In cases of proven soil contamination, public authorities could be allowed for intervention or even raise public awareness. The conflicts between public interest and privacy regarding land and in general environmental problems should be resolved at a legal basis.

The EIONET-CSI dataset will be supplemented with heavy metals data at European level. In 2009, 22,000 soil samples were taken in European Union countries during a soil survey named LUCAS [38]. Those soil samples have been analysed for some of the most important soil attributes such as soil organic carbon, and the results assist to estimate better the overall situation in Europe [39]. Currently, these soil samples are analysed for heavy metals, and the expected output results will facilitate better assessment of soil contamination in European Union. The LUCAS heavy metals dataset will face the issue of privacy which can overcome with the application of digital soil mapping for the development of interpolated maps. The combination of LUCAS heavy metals with EIONET-CSI will be an important step in assessing soil contamination in Europe.

The proposed datasets and the current study can be considered by public health professionals for epidemiological assessments. The study of human exposure pathways is a key issue on contaminated sites, and certainly the integration of EIONET-CSI datasets with epidemiological data would be a very important step forward in this direction. Moreover, as the majority of food is growing in soil, biomonitoring and other research should investigate the pathways and routes from producers to consumers.

## Figures and Tables

**Table 1:** Estimated and identified PCS and CS.

	Identified PCS and CS (a)	Estimated PCS (b)	Estimated CS (c)	Total (d)
Countries	33	12	11	38
Surveyed population	487,152,449	177,412,672	57,568,148	612,117,243
Surveyed surface area (km²)	4,460,305	1,552,984	833,188	5,772,075
Surveyed of total population	79.6%	29.0%	9.4%	
Surveyed of total area	77.3%	26.9%	14.4%	
PCS	1,169,649	739,968		2,553,000*
PCS/1000 capita	2.4	4.2		
CS	127,475		32,601	342,000*
CS/10,000 capita	2.62		5.7	
Remediated sites (RSs)	58,336			
RS/10,000 capita	1.20			

*Based on extrapolated data.

**Table 2:** Comparison of sectoral contribution to industrial contamination against the total number of enterprises.

Industrial/service sector	Sector contribution to industrial contamination (production)	Manufacturing sector	Number of enterprises (1,000)	Number of enterprises (1,000) contributing to 1% of industrial contamination
(a)	(b)	(c)	(d)	(e)
Chemical industry	8.2%	Chemicals plus rubber and plastic products	97.2	11.9
Metal working industry	13.1%	Basic metals plus fabricated metal products	381.2	29.1
Textile and leather industry	2.0%	Textiles plus wearing apparel plus leather	225.4	112.7
Wood and paper industry	3.7%	Wood and paper	191.8	51.8
Food industry and processing of organic products	5.7%	Food products plus beverages	273.8	48.0
Electronic industry	1.0%	Computer, electronic, plus electrical equip.	94.1	94.1
Mining sites	6.2%	Mining	18.2	2.9

Total	39.9%	Total	1281.7	
